# Dietary Management for Faecal Microbiota Transplant: An International Survey of Clinical and Research Practice, Knowledge and Attitudes

**DOI:** 10.3389/fnut.2021.653653

**Published:** 2021-10-25

**Authors:** Annabel K. Clancy, Anoja W. Gunaratne, Thomas J. Borody

**Affiliations:** Centre for Digestive Diseases, Five Dock, NSW, Australia

**Keywords:** faecal microbiota transplant (FMT), microbiome, dietary management, fibre, gastroenterology

## Abstract

Faecal microbiota transplantation (FMT) involves homogenisation and infusion of stool from a healthy, highly screened individual into the bowel of an unwell recipient. Dietary intake is an important modulator of the gut microbiota. Currently there are no clinical practice recommendations available to provide patients or stool donors with dietary advice for FMT. This study aimed to conduct an international survey to examine health professionals and researchers' attitudes, knowledge and current practice recommendations for diet in patients undergoing FMT. An online, cross-sectional, international survey comprising of health professionals and researchers managing patients undergoing treatment with FMT was conducted between July-October 2020. Purposeful and snowball sampling techniques were employed to identify eligible participants who were sent an email invitation and two email reminders with a link to participate in the electronic survey. The survey comprised 21 questions covering demographics, current practice, beliefs and future directions regarding FMT and diet. Closed responses were calculated as proportions of total responses. Open-ended responses were systematically categorised. Common themes were identified from recurring categories. Fifty-eight (M 60%) participants from 14 countries completed the survey. Participants were gastroenterologists (55%), with 1-5 years' experience working in FMT (48%) and treating up to ten patients with FMT per month (74%). Participants agreed that diet was an important consideration for FMT recipients and stool donors (both 71%), and that it would affect the outcomes of FMT. However, they did not feel confident in providing dietary advice to patients, nor that there was sufficient evidence to provide dietary advice and this was reflected in their practice. Future research must collect information on the dietary intake of patients and donors to better understand the relationship between diet and FMT outcomes. In clinical practice, promotion of healthy eating guidelines aligns with current practice and literature.

## Introduction

Faecal microbiota transplantation (FMT) is the process in which stool collected from a healthy, highly screened individual is homogenised, filtered and subsequently infused into the bowel of an unwell recipient. FMT aims to complement and re-establish more diversified microbiota within the gut of the recipient. Current FMT delivery methods vary, ranging from infusion into the caecum via colonoscope, to rectal enema infusions, and oral capsules (1). FMT treatment has shown marked success in the eradication of *Clostridium difficile* infection (CDI), with a cure rate of 96% reported in one randomised controlled trial (2). FMT has also shown promise in the treatment of chronic gastrointestinal conditions such as Ulcerative Colitis (UC), Crohn's Disease (CD) and Irritable Bowel Syndrome (IBS). A 2017 meta-analysis reported a pooled efficacy for clinical symptom remission of 36% in UC, and 52% for CD (3). Similarly, in IBS overall clinical response rate was 49% (4). FMT is also emerging as a potential treatment option for a variety of non-gastrointestinal conditions. For example, case reports have shown improvements in Parkinson's Disease (5), Multiple Sclerosis (6), Alopecia (7), Depression, and Anxiety (8).

Dietary intake, in particular dietary fibre is an important modulator of the gut microbiota composition. Fibres with fermentable characteristics are substrates for microbial populations in the colon, leading to the production of various metabolites. Dietary fibre interventions in healthy participants can influence bacterial abundance (9) and gut bacterial ecology (10). Subsequently it has been hypothesised that dietary patterns of donors and recipients could be a key predictor in the short and long term response to FMT treatment. However, currently dietary assessment of donors and recipients and the role of dietary interventions in supporting FMT has only just begun to be described (11, 12).

Pilot studies examining the relationship between recipient fibre intake and FMT outcomes have demonstrated a positive trend (13, 14), a study by Wei et al. compared the outcomes of UC patients receiving FMT and a known prebiotic fibre (pectin) supplement or FMT alone. Their results suggested that supplementation with pectin delayed the loss of diversity of transplanted gut microbiota and enhanced the effects of the FMT (15). Similarly, a study comparing the outcomes of patients with constipation receiving a soluble fibre supplement and FMT compared to FMT alone showed significantly improved stool frequency and consistency in the supplemented group in both the short and long term (16, 17).

Current consensus guidelines for donor screening do not include an assessment or requirement of specific diet for FMT donors (1, 18). One abstract study found FMT stool donors consumed higher fibre diets than the general American population (26 vs. 18 g/day) (19). More recently a focus on donors following specific dietary patterns such as veganism and Mediterranean diets (20) has emerged. For example, a trial of FMT treatment for metabolic syndrome utilising donor stool from a lean vegan donor demonstrated changes in recipient gut microbiota composition but not metabolic markers (21). Overall, despite a clear relationship between diet and the gut microbiome and FMT the gut microbiome, there is a distinct knowledge gap in the relationship between diet and FMT.

Currently there are no clinical practice recommendations available to clinicians to provide patients or stool donors with dietary advice for FMT. However, anecdotally, dietary advice is one of the most common questions a patient undergoing FMT treatment will ask for. In addition, as described above dietary patterns are hypothesised to be a key modulator of response to FMT treatment. Knowledge, attitude and practice surveys are a common tool used to collect information on what is known, believed and done in relation to a particular topic. These surveys are useful to establishing baseline practices and identifying needs, problems and barriers (22). Therefore, this study aimed to conduct an international survey to examine health professionals and researchers' attitudes, knowledge and current practice recommendations for diet in patients undergoing FMT.

## Materials and Methods

This study was an online, cross-sectional, international survey comprising of health professionals and researchers managing patients undergoing treatment with FMT.

Purposeful and snowball sampling techniques were employed to recruit a specific, yet professionally and geographically diverse sample of clinicians and researchers. Eligible participants were identified from professional FMT working group mailing lists, professional FMT network contacts, lead researchers of clinical trials in FMT identified on clinicaltrials.gov, clinicaltrialsregister.eu and the Australian and New Zealand Clinical trials register, clinics/hospitals/universities offering FMT treatment and lead authors on recently published FMT academic articles.

All eligible participants with available email addresses were sent an email invitation in July 2020 with a link to participate in the electronic survey, via the online survey tool, SurveyMonkey (23). The survey had 21 questions and took approximately 10 min to complete. The survey was designed by expert dietetic and FMT researchers and included questions on demographics, current practice, beliefs and future directions regarding FMT and diet (Table 1). Participation was anonymous and consent was implied on completion of the questionnaire. Participants were invited to share the survey invitation and link with professional contacts. A reminder email was sent at 1 and 2 months after the initial invitation and the survey was closed in October 2020 after being open for 3 months. Ethics approval was obtained from the institutional Human Research Ethics Committee (CDD20/C03).

**Table 1:** Survey questions.

1. What is your age?
2. What is your gender?
3. What is your country of practice?
4. What is your highest level of education?
5. What is your profession? (Please choose all that apply)
6. How many years experience do you have working in the field of FMT?
7. How many patients per month do you treat with FMT?
8. For which indications do you treat patients with FMT? (Please choose all that apply)
9. Please indicate your level of agreement or disagreement with the following statements: • Dietary intake will impact an individual's response to FMT • It is important to consider dietary intake of patients undergoing FMT • I feel confident providing dietary recommendations to patients undergoing FMT • It is important to consider the dietary intake of donors providing stool for FMT
10. Do you gather dietary information from patients undergoing FMT?
11. Do you provide dietary advice or recommend patients follow a specific diet prior to FMT?
12. Do you recommend any dietary supplements for patients prior to FMT?
13. Do you provide dietary advice or recommend patients follow a specific diet post FMT?
14. Do you recommend any dietary supplements for patients post FMT?
15. Do you recommend patients see a dietitian/nutritionist pre or post FMT?
16. Do you provide dietary recommendations or recommend stool donors follow a specific diet during their donation period?
17. Do you recommend any dietary supplements for stool donors?
18. What do you feel, if any, are the facilitators to effective dietary management of patients undergoing FMT?
19. What do you feel, if any, are the barriers to effective dietary management of patients undergoing FMT?
20. In the dietary management of patients undergoing FMT, what are for you the most important problems or unanswered questions?
21. Do you have any other comments or suggestions?

Data was exported directly into an Excel database from the electronic survey. Closed responses were calculated as proportions of total responses and presented as percentages. Open-ended responses were systematically examined and categorised. Common themes were identified from recurring categories. Where possible, common themes were also calculated as proportions of total responses to provide a broader understanding of closed responses.

## Results

A total of 380 potential participants with available email addresses were identified and contacted. Fifty-eight responses were received, providing an estimated participation rate of 15%.

Participants were more likely to be male, aged 31-40 years with a medical degree. Of the participants from 14 countries, approximately half of respondents were practicing as gastroenterologists and resided in Australia or the United States of America (USA). Most participants reported between 1 and 5 years' experience working in FMT and treating up to ten patients with FMT per month. CDI was the most common indication participants treated with FMT, followed by UC (Table 2).

**Table 2:** Demographics of the participants (*n* = 58).

**Characteristic**	**Number (%)**
**Age (years)**	
21-30	4 (7)
31-40	23 (40)
41-50	13 (22)
51-60	12 (21)
61-70	6 (10)
70+	0 (0)
**Gender**	
Male	35 (60)
Female	22 (38)
Other	1 (2)
**Country of practice**	
Australia	15 (27)
Belgium	2 (4)
Canada	5 (9)
China	2 (4)
Czechia	1 (2)
Denmark	1 (2)
France	1 (2)
Germany	2 (4)
Hungary	1 (2)
Israel	3 (5)
Norway	1 (2)
Russia	1 (2)
United Kingdom	6 (11)
United States of America	14 (25)
No response	3 (5)
**Highest level of education**	
Bachelor/undergraduate degree	9 (16)
Master's degree	6 (10)
Doctoral degree (MD)	24 (41)
Doctoral degree (PhD)	15 (26)
Doctoral degree (ND)	2 (3)
Other	2(3)
**Profession**	
Gastroenterologist (Adult)	27 (47)
Gastroenterologist (Paediatric)	5 (9)
Nurse	7 (12)
Research assistant	1 (2)
Dietitian/nutritionist	4 (7)
General practitioner	1 (2)
PhD candidate/Doctoral student	5 (9)
Postdoctoral researcher	3 (5)
Mid-career researcher/Senior research fellow	4 (7)
Leading researcher/Professor	3 (5)
Oncologist	1 (2)
Infectious diseases physician	3 (5)
Naturopath	1 (2)
Other	4 (7)
**Experience in FMT (years)**	
None	1 (2)
1-5	28 (48)
6-10	18 (31)
11-15	7 (12)
16-20	4 (7)
20+	0
**Number of patients treated per month**	
None	6 (10)
1-10	43 (74)
11-20	6 (10)
21-30	1 (2)
31-40	0 (0)
41-50	1 (2)
51+	1 (2)
Indications treated with FMT by participants^*^	
*Clostridium difficile* infection	47 (81)
Ulcerative colitis	23 (40)
Crohn's disease	12 (21)
Irritable bowel syndrome	13 (22)
Multi-dug resistant organisms	5 (9)
Autism spectrum disorder	2 (3)
Cancer	3 (5)
Graft vs. host disease	2 (3)
Cirrhosis	2 (3)
Other	7 (12)

* *Proportion of participants who indicated they treated each indication. Participants could provide more than one response*.

Participants were asked to provide their level of agreement or disagreement with statements relating to diet and FMT (Table 3). Overall, two thirds (66%) of participants agreed that diet will impact an individual's response to FMT. They also agreed that the diet of both patients undergoing FMT and stool donors should be considered (both 71%). Interestingly, less than half (42%) of participants indicated that they felt confident providing dietary recommendations to patients undergoing FMT.

**Table 3:** Participants attitudes and beliefs regarding diet and FMT (*n* = 58).

**Statement**	**Strongly disagree** ***N* (%)**	**Disagree** ***N* (%)**	**Neutral** ***N* (%)**	**Agree** ***N* (%)**	**Strongly agree** ***N* (%)**
Dietary intake will impact an individual's response to FMT	4 (7)	3 (5)	13 (22)	30 (52)	8 (14)
It is important to consider dietary intake of patients undergoing FMT	5 (9)	2 (3)	10 (17)	26 (45)	15 (26)
I feel confident providing dietary recommendations to patients undergoing FMT	7 (12)	10 (17)	17 (29)	16 (28)	8 (14)
It is important to consider the dietary intake of donors providing stool for FMT	2 (3)	2 (3)	13 (22)	26 (45)	15 (26)

Participants were also asked about their current clinical/research practice for patients undergoing FMT. Half of the participants reported (54%) that they did not collect dietary information from patients undergoing FMT. Of those that did report collecting dietary information (46%), this was often under a clinical trial setting, and varied from “type of diet,” to food frequency questionnaires or food diaries. Participants did not routinely recommend patients see a dietitian/nutritionist before or after FMT (69%). Reasons included dietitians being unfamiliar with FMT, nurses or other team members providing the nutrition information, or only if indicated for other circumstances such as a restricted diet or malnutrition.

More specifically, current clinical/research practice for patients prior to and post FMT was examined. Close to three quarters (72%) of participants reported that they did not provide any dietary advice or recommend recipients a specific diet prior to FMT. Of those that did provide dietary advice before FMT (28%), most recommended a high fibre/high prebiotic/high fruit and vegetable diet (10%). Conversely, a low fibre/low FODMAP diet was also recommended (9%) as was maintenance of diet (3%). The majority (91%) of participants did not suggest dietary supplements prior to FMT. Of those that did recommend supplements (9%), fibre supplements were the most commonly recommended (5%).

Current clinical/research practice reported by participants for patients after FMT was similar to pre FMT. Just over half (57%) of participants did not recommend patients follow a specific diet after FMT. Of the 43% who did provide dietary recommendations, the majority (33%) focused on healthy eating guidelines and/or a high fibre diet. Fermented foods and dietary changes for symptom management were also recommended (both 5%). Dietary supplements after FMT were not routinely recommended (84%). Ten percent of participants recommended patients take a fibre supplement after FMT. Two participants suggested probiotic supplements and one promoted butyrate supplements.

Current dietary recommendations in clinical/research practice for stool donors was also examined. The majority (60%) of participants did not provide dietary recommendations or require a specific diet for stool donors. Dietary recommendations that were provided to stool donors included healthy eating guidelines and/or high fibre diet (22%), avoidance of processed foods, or avoidance of raw or risky foods, especially meats and seafood (both 9%) and avoidance of common food allergens or foods recipients are allergic to (7%). Dietary supplements were not recommended for stool donors (96%).

Facilitators and barriers to dietary management in FMT were explored through open ended questions. Facilitators identified included; collaboration with a clinical nutrition professional, and effective communication and dietary education of patients. Two common themes surrounding barriers to effective dietary management of patients undergoing FMT were identified. Firstly, lack of available evidence and research to support the role of diet in FMT. This also included recognition of the provider's lack of knowledge. Secondly, the limited role or lack of access to dietitians/nutritionists and a lack of dietary education resources. Participants also reported time constraints, poor patient compliance due to apathy or difficulty coping with dietary change physically (symptoms) or emotionally and specificity of patient's dietary requirements (e.g., food intolerances) as barriers.

Two main areas for further research were identified by participants. Firstly, the importance of a patients diet in the success of FMT. It was noted that this would need to be indication specific, with one participant suggesting a limited role of diet in CDI. There was also the question of whether dietary preparation, e.g., fasting prior to FMT was important. Secondly, the importance of, and the best diet for donors for the success of FMT.

## Discussion

To the best of our knowledge this is the first study to examine health professionals and researchers' attitudes, knowledge and current practice recommendations for diet in patients undergoing FMT. Overall, health professionals and researchers in this group agreed that diet was an important consideration for FMT recipients and stool donors, and that it would affect the outcomes of FMT. However, they did not feel confident in providing dietary advice to patients, nor that there was sufficient evidence to provide dietary advice and this was reflected in their practice.

Overall, dietary advice was not routinely provided to patients before or after FMT. Current research regarding the role of a patient's diet and dietary supplements in FMT is limited. One animal study has demonstrated that initially, post FMT, the microbial profile of mouse stool was aligned with their donor, irrespective of diet. However, by week 22, the faecal microbiome profile no longer reflected the donor but could be differentiated by the diet (24). In a pilot study in humans, a diet higher in fruit and fibre was associated with successful treatment outcomes in CDI (13). Similarly, remission was achieved in a case report of severe UC treated with FMT and a low sulphur, high fibre diet (25). In addition, dietary supplementation with fibre has been shown to enhance clinical outcomes of participants receiving FMT for UC and constipation compared to those receiving FMT alone (15, 16). Overall, dietary data is not currently routinely collected or reported in FMT studies. Generating information on patients undergoing FMT and their diets should start by establishing baseline diets of patients undergoing FMT. Subsequently, associations identified between diet and participant outcomes for specific indications could be used to design animal studies and human intervention trials.

The limited available data in this area was reflected in our cohort's dietary advice with the majority of participants not providing dietary advice or recommending dietary supplements. Conversely, results from this survey indicated that when dietary advice was provided the patients, it was consistent with the current available evidence (13, 15, 16) and healthy eating guidelines (26). Globally, healthy eating guidelines promote fruit and vegetable consumption and limit alcohol, highly processed foods and beverages (26). These guidelines are backed by high quality evidence, align with prevention or remission guidelines for most chronic health conditions, are simple and promote no risk to patients (27–29). Evidence also suggests that the general population (30–32) and patients with gastrointestinal conditions rarely meet these guidelines (33–35). Therefore, a high fibre diet, promoting fruit and vegetable consumption, in line with healthy eating guidelines could be advised for patients undergoing FMT.

Other reasons for the lack of dietary advice provided to patients undergoing FMT was a lack of access to suitably trained dietitians, time constraints, poor patient compliance and dietary requirements. Therefore, upskilling physicians and dietitians in this area is important to ensuring quality of care. Dietitians are health professionals with specific knowledge and training in the application of the science of food and nutrition to improve the health outcomes of individuals (36). Many participants indicated that including a clinical nutrition professional in the care of patients undergoing FMT was an important facilitator to effective dietary management of these patients. Certainly, including a dietitian in the care of patients undergoing FMT may overcome many of the barriers identified by participants. For example, including a dietitians can assist with patient compliance and ensure tailored advice for specific dietary restrictions (37–39). Conversely, some participants indicated that dietitians were unfamiliar with FMT, indicating a need for further training in this area. However, if the current dietary advice that should be provided to patients is in line with national dietary guidelines then dietitians are well-equipped to support patients (36, 40). Overall, including a dietitian in the care of patients with FMT will likely provide benefit. Increasing accessibility to dietitians will likely be country specific and should also be considered. Training and upskilling of dietitians and health care providers in FMT and the development of specific FMT dietary education resources are also considerations for the future.

Results from this study indicated that dietary advice was not routinely provided to FMT stool donors. This is in line with a recent systematic literature review and meta-analysis of donor screening procedures (41) and current consensus guidelines for FMT production, which focus on potential pathogen transmission (1, 18). In our study, if donors were provided with dietary advice, a high fibre diet in line with healthy eating guidelines was recommended. Interestingly, one participant indicated that a number of their donors were vegetarian. Whilst, another suggested that donors already had healthy dietary habits. A study from the United States examined diets of donors and identified that their diet was similar to the general American population, although higher in fibre (26g vs. 18g) (19). However, this is still below recommended guidelines (42). The importance of diet and the best diet for donors still remains to be elucidated. Evidence from animal models suggests that the FMT from a donor with a high fibre diet promoted greater improvements in emphysema compared to diet or FMT alone (43). Increasing evidence regarding the impact of stool donor microbial composition on patient outcomes also suggests that diet will have an important role. For example, donor stool rich in *bifidobacterium*, known to be enhanced by a fibre rich diet (44), was associated with increased therapeutic efficacy of FMT in patients with IBS (45). Furthermore, a recent study examining the effects of diet-modulated autologous FMT in preventing weight regain has shown the potential for specific dietary patterns to attenuate weight regain (20). However, diet as a donor selection criterion will need to be carefully balanced with donor recruitment procedures, which are already highly restrictive (46). Researchers should incorporate dietary data collection from stool donors into their studies in order to generate greater knowledge in this area.

Overall, this study met the aim of conducting an international survey and examining attitudes, knowledge and current practice by health professionals and researcher's for diet in patients undergoing FMT. Results establish a baseline practice of dietary recommendation for patients undergoing FMT and donors. The survey design was beneficial in enabling views of participants globally to be captured and the participation rate was in line with physician surveys (47). However, it is recognised that there is a potential for response bias and that viewpoints may have been missed. A number of participants also reported limited experience in FMT treatment which may have also bias the results. Additionally, the survey questions did not discriminate dietary advice by treatment indication. However, given that three-quarters of respondents did not provide dietary advice it is unlikely that this would influence the results. Overall, it is clear that further research into the role of donors and recipients diets is of interest and importance. Clinicians and researchers should start by establishing baseline diets of patients undergoing FMT for all indications. Combined FMT and dietary intervention studies for UC, constipation and obesity should also be conducted. Incorporation of dietary intake data collection of donors into research studies is also recommended.

This study examined health professionals and researchers' attitudes, knowledge and current practice recommendations for diet in patients undergoing FMT. Health professionals and researchers in this group agreed that diet was an important consideration for patients and stool donors, and that it would influence the outcomes of FMT. However, in practice, dietary advice was not provided to patients as they did not feel confident in providing dietary advice, nor that there was sufficient evidence to provide dietary advice. Future research must collect information on the dietary intake of patients and donors to better understand the relationship between diet and FMT outcomes. In clinical practice, promotion of healthy eating guidelines especially fruit and vegetable consumption aligns with current practice and literature and can be encouraged for patients undergoing FMT and stool donors.

## Data Availability Statement

The original contributions presented in the study are included in the article/supplementary material, further inquiries can be directed to the corresponding author/s.

## Ethics Statement

The studies involving human participants were reviewed and approved by Centre for Digestive Diseases Human Research Ethics Committee. The patients/participants provided their written informed consent to participate in this study.

## Author Contributions

AC was responsible for the study design, data collection, data analysis, and drafting of the manuscript. AG was responsible for support of study design, data analysis, and manuscript drafting. TB was responsible for support of study design, data analysis, and manuscript drafting. All authors contributed to the article and approved the submitted version.

## Conflict of Interest

TB has a pecuniary interest in the Centre for Digestive Diseases, is a medical advisor to Finch Therapeutics Inc. and holds patents in FMT treatment. The remaining authors declare that the research was conducted in the absence of any commercial or financial relationships that could be construed as a potential conflict of interest.

## Publisher's Note

All claims expressed in this article are solely those of the authors and do not necessarily represent those of their affiliated organizations, or those of the publisher, the editors and the reviewers. Any product that may be evaluated in this article, or claim that may be made by its manufacturer, is not guaranteed or endorsed by the publisher.
